# A randomised controlled trial to determine the effect on response of including a lottery incentive in health surveys [ISRCTN32203485]

**DOI:** 10.1186/1472-6963-4-30

**Published:** 2004-11-08

**Authors:** LM Roberts, S Wilson, A Roalfe, P Bridge

**Affiliations:** 1 Dept. Primary Care and General Practice, Division of Primary Care, Public and Occupational Health, The University of Birmingham, Edgbaston, Birmingham, B15 2TT, UK

## Abstract

**Background:**

Postal questionnaires are an economical and simple method of data collection for research purposes but are subject to non-response bias. Several studies have explored the effect of monetary and non-monetary incentives on response. Recent meta-analyses conclude that financial incentives are an effective way of increasing response rates. However, large surveys rarely have the resources to reward individual participants. Three previous papers report on the effectiveness of lottery incentives with contradictory results. This study aimed to determine the effect of including a lottery-style incentive on response rates to a postal health survey.

**Methods:**

Randomised controlled trial. Setting: North and West Birmingham. 8,645 patients aged 18 or over randomly selected from registers of eight general practices (family physician practices). Intervention: Inclusion of a flyer and letter with a health questionnaire informing patients that returned questionnaires would be entered into a lottery-style draw for £100 of gift vouchers. Control: Health questionnaire accompanied only by standard letter of explanation. Main outcome measures: Response rate and completion rate to questionnaire.

**Results:**

5,209 individuals responded with identical rates in both groups (62.1%). Practice, patient age, sex and Townsend score (a postcode based deprivation measure) were identified as predictive of response, with higher response related to older age, being female and living in an area with a lower Townsend score (less deprived).

**Conclusion:**

This RCT, using a large community based sample, found that the offer of entry into a lottery style draw for £100 of High Street vouchers has no effect on response rates to a postal health questionnaire.

## Background

Self-completed postal questionnaires are an economical and simple method of data collection for both research studies and audit activity. They are cheaper than telephone or personal interviews [1] and may be particularly useful in medical research as the response rate to sensitive questions may be greater than from other methods of data collection [2]. However, questionnaire based studies are subject to non-response bias [3]. If there are differential response rates from certain groups in the sample population, then generalisability of results to the target population is questionable. The lower the response rate, the more open to criticism the conclusions drawn from the research will be. High response rates are important not only because they reduce the risk of non-response bias but also because they increase the precision of parameter estimates [4]. Ensuring a high response rate to initial mailings has the additional benefit of reducing the costs associated with re-mailing or other methods of follow-up such as telephone interviews.

Strategies for increasing response rates can be summarised as falling into five broad categories; the covering letter (personalisation, use of an appeal etc), incentives (cash or other reward), contact (pre-notification and follow-up), mailing (return envelopes, type of outgoing postage etc) and questionnaire (length, format, colour etc) [5]. Pre-notification, follow-up, university sponsorship, cash incentives, first class postage, freepost return and questionnaire colour have all been shown to increase response rates [4]. A Cochrane review [3,6] reported similar findings, with monetary incentives and recorded delivery having the greatest effect on response (response rates doubled) and a range of other strategies significantly increasing the odds of response. However, individual studies demonstrated wide variability, were conducted across a variety of disciplines (psychology, medicine, business etc) and in a variety of countries; their generalisability is, therefore, questionable. An individual's decision to reply to a questionnaire is bound up in the content of the questionnaire, its relevance to the individual and who is perceived as benefiting from completion [3]. Questionnaires asking about personal experience may be treated differently to those asking for opinion, and information requested by a retail company may be perceived as less important than that sought by a charity, academic institution or health provider.

Much of the research relating to the effectiveness of specific interventions on response rates in general population surveys was conducted before 1990 and from a market or sociological perspective. More recent work has been conducted on the effects of incentives on increasing response rates in populations of medical professionals. A lottery style incentive (prize of a weekend break) was reported to generate a small increase in response rates from General Practitioners (GP, Family physicians) in Quebec [7] and cash incentives also increased response rates in a GP population [8]. It is not clear, however, whether results from these professional groups can be generalised to general population surveys. Studies sampling members of the public tend to confirm the more general findings of the Cochrane review [6], which included surveys of professionals as well as the public, that financial incentives have a positive effect on response and even a small financial incentive ($1 – $2) increased response rates to a health related survey [9,10]. However general population surveys often require large samples and the cost of offering even a small individual monetary reward may be prohibitive.

The Cochrane Review [6] identified 45 trials (44,708 participants) of non-monetary incentives (e.g. key ring, offer of entry into a lottery, offer of study results) and found that the odds of response were approximately a fifth higher when using such incentives. However, their meta-analysis did not discriminate between the type of non-monetary incentive, the population (i.e. professional versus public) or the subject matter (i.e. health versus non-health). Of the nine studies identified that utilised some form of lottery incentive [6], only one demonstrated a significant benefit [11]. The remaining eight studies had non-significant results although five of these suggested a trend towards an improved response [12-16], two tended to suggest a detrimental effect [17,18] and one showed no effect [19]. Evidence on the use of lottery style incentives is therefore conflicting. One of the studies that showed no overall benefit in including a lottery incentive [13] demonstrated a significant difference in favour of the lottery group (24.4% versus 18.5%) with respect to responses to a first mailing. Although this benefit was not maintained after a reminder, such a result could reduce the cost of undertaking surveys.

Three of the five [13,15,16,18,19] evaluations of a lottery incentive using a general population sample, indicated small but non-significant increases in response. The three studies that demonstrated a tendency towards increased response rates had offered entry into a draw for a restaurant meal to the value of $100 (1985) [15], $50–$200 (1988) [16], and a range of prizes including a television, $100 and a trip to Las Vegas (1989) [13]. There is, therefore, some evidence to support the use of lottery incentives in increasing questionnaire response rates. However, studies focussing on health related issues are limited to only two that used a general population sample [15,19] and three that sampled patients [12,14,17]. The wide range of incentives used, study settings and subjects mean that it is difficult for medical researchers to determine the applicability of this evidence to community based surveys in the UK.

Determining the community prevalence of disease is important for needs assessment, service planning and determining the potential economic implications of new treatments. Studies aiming to precisely estimate the prevalence of disease usually require large samples and often use postal questionnaires. A large community mailing to determine the prevalence of Irritable Bowel Syndrome (IBS) [20] provided an opportunity to embed a randomised controlled trial to investigate the effect of including an incentive on response and completion rates. Medical research rarely, unless sponsored by the pharmaceutical industry, has the capacity to pay a cash incentive for all returned questionnaires. This study therefore aimed to determine the effect of including a low-cost lottery style incentive (returned questionnaires being entered into a draw for a prize) on the response and completion rate of a health questionnaire survey. A secondary objective was to combine data from this study with that from previously published studies based in a health environment [15,19] using patient/general population responders to provide a more precise estimate of effect.

## Methods

### Location

This study was undertaken in Birmingham, a large city in the West Midlands region of the United Kingdom. Ethical approval was obtained from North Birmingham and West Birmingham Research Ethics Committees prior to commencement of the study.

### Participants

Eight general practices in the North and West Birmingham areas were recruited to participate in a health survey designed to determine the prevalence of IBS in the community [20]. All practices in these areas were invited to participate in this survey and practices were randomly selected from those practices who had expressed an interest in participation, after stratification for deprivation scores. To provide a sample with representation from all socio-economic groups, practices were selected from each of the four quartiles of the Townsend scores. Townsend scores are calculated from small area statistics collected during the decennial census (most recent in 2001) and provide an indicator of deprivation. Practices were allocated a Townsend score based on their location (postcode). All patients aged 18 and over were eligible for inclusion. The only exclusion criteria were patients for whom the general practitioner indicated that mailing would be inappropriate; this typically included patients known to be terminally ill or patients unable to complete the questionnaire e.g. those with severe learning disability. Questionnaires returned by the postal service as 'not at this address' were removed from the sampling frame (denominator) and response rates calculated as the number returned divided by this denominator.

### Trial size

Estimates were based on the prevalence study within which this trial was embedded and indicated that 8,000 patients should be mailed. This provided 4,000 in each arm of the RCT, sufficient to demonstrate a 4% difference in response rates with 90% power at the 5% significance level, assuming a 60% response rate.

### Randomisation

To minimise contamination (two or more individuals in a household receiving a questionnaire, but not all receiving the lottery incentive) only one person per domestic address was selected. Practice registers were utilised to generate randomly ordered lists of addresses and then one individual aged 18 or over was randomly selected from each address until the required number of patients had been identified. The prevalence survey, within which this trial was embedded, aimed to recruit a stratified random sample, comprising 1,150 patients from each participating practice to ensure sufficient cases were included from each of the Townsend quartiles. Where the practice was unable to generate 1,150 patients (due to smaller list sizes) the maximum number of patients available was included. Randomisation was performed on a 50:50 basis within each practice, to control for practice effects, using a computerised simple random number sequence.

### Intervention

All patients selected received a health questionnaire. Questionnaires had three sections; section one requested personal and demographic details in addition to details of personal and family medical history; section two was the SF12 [21], a validated generic quality of life measure; section three was a self completed questionnaire version of the ROME II criteria [22] (to confirm diagnosis of IBS). A covering letter, sent in the joint names of the University and the relevant general practice, explained that the practice was participating in a research project to find out about the number of patients affected by certain conditions and the ways in which ill health affects people's quality of life. All patients received a reply paid envelope (addressed to the research team at the University of Birmingham) with the questionnaire and were informed that return of a blank questionnaire would indicate the wish not to be involved and they would not be contacted further.

The intervention group received an identical questionnaire to the control group. The covering letter was also identical apart from the addition of a paragraph explaining that all returned questionnaires would be entered into a draw for a prize of £100 of "High Street shopping vouchers". This letter stressed that entry into the draw was dependent on return rather than completion of a questionnaire, as this was deemed to be more ethically responsible. In addition to the letter, a flyer printed on brightly coloured paper (yellow) was included for intervention patients, highlighting the fact that returned questionnaires would be entered into a draw.

All non-responders were re-mailed after 4 weeks. Again, the intervention group received the additional paragraph in the covering letter and an additional flyer. All follow-up mailings included a copy of the questionnaire and a reply paid return envelope. Data handlers were not blinded to the intervention status of responders, but this was not considered to be a source of bias as response rates were the primary outcome.

Mailings took place in the period January to July 2001. Mailings were conducted by practice and within each practice, control and intervention patients were mailed on the same day.

### Outcomes

The principal outcome was the overall response rate. Response rates to initial and follow-up mailings, and numbers of blank responses were also compared between groups.

### Analysis

Analysis was on an intention to treat basis. Response rates were compared between the two arms of the trial using chi-squared tests. Predictors of response were identified by logistic regression using backward elimination. Variables entered into the starting model included randomisation arm, age, sex, Townsend score derived from patient postcodes, practice and all two-way interactions with the randomisation arm. Two recent systematic reviews of strategies to influence response rate were identified [3,4] and from these, all studies of non-professional groups using lottery-style incentives in a health environment were identified and full papers obtained. Data from these was combined with those of this study and a meta-analysis undertaken (Rev Man software).

## Results

Eight thousand six hundred and forty five patients were included in the trial; 4,325 were randomised to the lottery arm and 4,320 to the control group and a 50:50 split was maintained within each practice.

**Figure 1:**
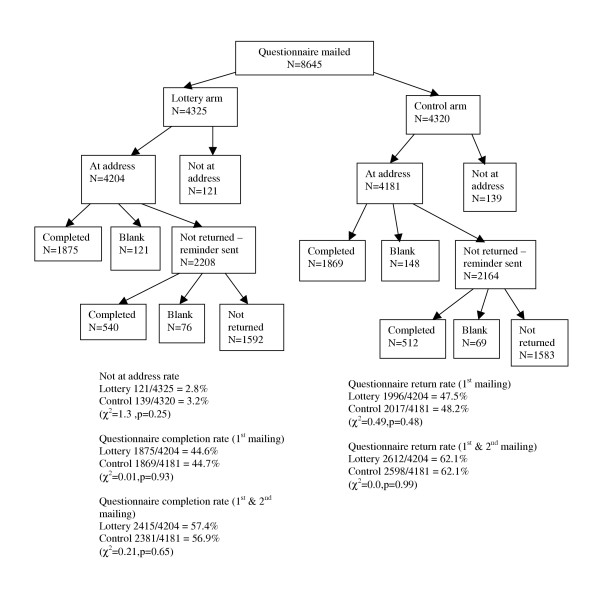
Questionnaire response rates

The trial profile is shown in Figure 1. Two hundred and sixty questionnaires were returned by the Royal Mail as 'not at address' and were not included in the analysis. The proportion of these returned questionnaires were comparable between trial arms (121 (2.8%) in the intervention arm and 139 (3.2%) in the control arm). Baseline characteristics of patients were similar between the two randomised groups (Table 1).

Four thousand and twelve individuals responded to the initial mailing; 1996 (47.5%) in the intervention arm and 2017 (48.2%) in the control group (χ^2 ^= 0.5, p = 0.48). A further 1197 replied to the reminder mailing; 616 in the intervention arm and 581 in the control arm giving an overall response of 2612/4204 (62.1%) in the intervention arm and 2598/4181 (62.1%) in the control arm (χ^2 ^= 0, p = 0.99). The numbers of questionnaires returned blank was similar for both groups, 197 (4.7%) in the intervention and 217 (5.2%) in the control arm (χ^2 ^= 1.1, p = 0.29). No objections to the use of an incentive or requests for exclusion from the prize draw were made to the research team or participating general practices.

Response rates varied by practice from 37% to 77% (Table 2). Practice, patient age, patient sex and Townsend score were identified as predictive of response, with response rates increasing with age ((OR per 1 year change in age) (95% CI) = 1.02 (1.016, 1.024)) (i.e. if the odds of responding at age 40 are 1.0 (1:1), then odds of responding at age 45 are 1.1 (1:1.1)). Lower response rates were associated with being male (OR = 0.53 (0.48, 0.58)) and living in an area with a higher Townsend score (more deprived) (OR per 1 point change in deprivation score= 0.93 (0.92, 0.95) (Table 3). The small number of practices, and limited number of explanatory variables associated with each practice, meant that whilst practice was predictive of response it was not possible to explore the practice characteristics related to this. Randomisation arm and its interaction with other terms were not identified as significant predictors of response rate. This indicates that practice, patient age, sex and Townsend score affected response rates whereas the lottery incentive did not.

Given the lack of difference between groups, no economic evaluation was conducted. The lottery incentive presented additional costs in terms of production and inclusion of flyers (<£0.01 per individual) and provision of the pre-specified prize (£100 overall), and there was therefore a net disbenefit to the use of the incentive.

**Figure 2:**
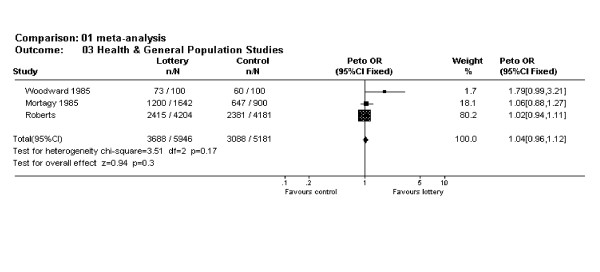
Meta-analysis of lottery studies using health related general population surveys

Two previously published relevant studies were identified [15,19]. Results of the meta-analysis are provided in Figure 2.

## Discussion

This RCT using a large community sample suggests that using a lottery draw style incentive for £100 of High Street vouchers has no effect on overall response rates to a postal questionnaire asking about health and medical history. Whilst it is possible that the locality of the study (Birmingham, UK) is not typical, the range of practices and Townsend scores of the sample, suggest this is unlikely. It is also possible that a larger incentive or a cash prize, rather than vouchers, may influence response rates. However the value of the incentive offered was comparable to, or greater than, previous studies. The only published study which has shown a significant benefit of a prize draw was of professionals [11] and we, therefore, believe that the findings of this large trial are likely to be generalisable to health related surveys of the general public.

There was no difference between the groups in the numbers of questionnaires returned blank indicating that the incentive did not just encourage people to return incomplete questionnaires. There was also no difference in the numbers replying to the first mailing. Had the incentive increased numbers replying to the initial mailing, even if it failed to increase response overall, it may have proved cost-effective in terms of reducing the costs of follow-up mailings. Our results indicate that previous research suggesting increased response to initial mailings [13] is not generalisable.

The questionnaire used in this RCT was designed to determine disease prevalence. It is possible that such mailings over-inflate prevalence estimates, as individuals responding may be encouraged to do so because of their vested interest as an individual experiencing disease. Inclusion of an incentive not related to disease status may encourage response from a wider range of the population and may provide more accurate estimates of prevalence. However, this study does not suggest that future health surveys would gain any response benefit from the inclusion of a prize draw incentive. Indeed the addition of the data from this study to that of the two previously published studies based in a health environment [15,19] using patient/general population responders provides further evidence of a lack of effect (Figure 2). Future work in this area may best be conducted using qualitative methodologies to explore factors related to response in community surveys.

## Conclusions

A lottery draw style incentive for £100 of High Street vouchers does not affect response rates to a postal health survey, when used in a general population sample. On the basis of this large RCT we would not recommend utilising similar incentives in general population health research.

## Competing interests

The author(s) declare that they have no competing interests.

## Authors' Contributions

SW and LR conceived the idea and designed the study. PB contributed to the study design and collected and validated the data. AR completed the analyses. LR wrote the first draft of the manuscript, all other authors made significant contributions to the writing of the manuscript and approved the final version. SW is the guarantor.

## Pre-publication history

The pre-publication history for this paper can be accessed here:



## Figures and Tables

**Table 1:** Baseline characteristics of patients by randomisation arm

Characteristic		Lottery	Control
Age (years)	mean (sd)	48.5 (18.8)	48.4 (18.8)
	range	18–100	18–98
	n	4323	4316
Sex	(male) %	47.8	48.2
	n	4325	4320
Townsend score*	mean (sd)	1.06 (3.6)	1.06 (3.7)
	median (IQR)	1.9 (-2.25 to 4.3)	1.9 (-2.11 to 4.3)
	n	4199	4189

Townsend scores are calculated at enumeration district level and standardised to England and Wales (Range -7.6 to 11.8). Positive scores indicate greater deprivation than the national average.

**Table 2:** Response rates

	Lottery			Control			Total
	
Practice	Number Responded	Total mailed	%	Number Responded	Total mailed	%	%
1	365	558	65.4	359	557	64.5	64.9
4	394	555	71.0	390	547	71.3	71.1
5	333	522	63.8	335	519	64.6	64.2
8	423	553	76.5	426	556	76.6	76.6
3	329	538	61.2	318	547	58.1	59.6
2	371	558	66.5	355	553	64.2	65.4
6	137	367	37.3	132	353	37.4	37.4
7	259	553	46.8	283	549	51.5	49.2

**Table 3:** Factors related to response.

Variable	β (SE)	P value	Odds ratio (95% confidence interval)
*Intercept*	-0.36 (0.06)	<0.0001	-
Age (per increase of 1 year)	0.02 (0.001)	<0.0001	1.02 (1.02, 1.02)
Male	-0.64 (0.05)	<0.0001	0.53 (0.48, 0.58)
Townsend score (per increase of 1 unit)	-0.07 (0.01)	<0.0001	0.93 (0.92, 0.95)
Practice 3	-0.22 (0.07)	<0.0001	0.80 (0.69, 0.92)
Practice 6	-0.90 (0.09)	<0.0001	0.41 (0.34, 0.48)
Practice 7	-0.67 (0.07)	<0.0001	0.51 (0.45, 0.59)

Variables considered were randomisation arm, age, sex, Townsend score, practice and 2 way interactions with randomisation arm.
